# Microbiological risk infection assessment using QMRA in agriculture systems in Côte d’Ivoire, West Africa

**DOI:** 10.1007/s10661-017-6279-6

**Published:** 2017-10-28

**Authors:** Parfait K. Kouamé, Hung Nguyen-Viet, Kouassi Dongo, Christian Zurbrügg, Jean Biémi, Bassirou Bonfoh

**Affiliations:** 1 Unité de Formation et de Recherche des Sciences de la Terre et des Ressources Minières, UFR-STRM, Université Félix Houphouët-Boigny, 22 BP 582, Abidjan, 22 Côte d’Ivoire; 2 Centre Suisse de Recherches Scientifiques en Côte d’Ivoire, 01 BP 1303, Abidjan, Côte d’Ivoire; 3 International Livestock Research Institute (ILRI), Room 301-302, B1 Building, Van Phuc Diplomatic Compound 298 Kim Ma Street, Ba Dinh District, Hanoi, Vietnam; 4 Eawag, Swiss Federal Institute of Aquatic Science and Technology, Department of Water, Sanitation and Solid Waste for Development, Überlandstrasse 133, 8600 Dübendorf, Switzerland; 5 Swiss Tropical and Public Health Institute, Swiss TPH, Socinstrasse 57, 4051 Basel, Switzerland

**Keywords:** QMRA, Health risk, Agriculture system, Wastewater, Surface water, Côte d’Ivoire

## Abstract

Poor wastewater management that results from a lack of appropriate sanitation infrastructure contributes to increasing health risks in urban areas in Côte d’Ivoire. We assessed the health risks associated with the use of wastewater for watering salad destined for human consumption, to help local authorities in developing appropriate risk mitigation measures for Yamoussoukro, the political capital of Côte d’Ivoire. We applied a stochastic approach based on quantitative microbiological risk assessment (QMRA), focusing on wastewater for farming activities and salad consumption at the household level. Farming activities rely on a large degree on contaminated water and are conducted without any protection. The QMRA highlights that the poor quality of watering water increased the microbiological risk of the two assessed groups of urban farmers and individual households. The annual risk of infection due to watering wastewater in the city is estimated at 0.01 per person per year (pppy) for *Giardia lamblia* and 0.2 pppy for *Escherichia coli* O157:H7. The annual risk from salad consumption is 0.01 pppy for *G. lamblia* and 0.9 pppy for *E. coli* O157:H7. Both the annual risks from farming activities and salad consumption were higher than the tolerable standard of risk of 10^−4^ pppy as defined by the World Health Organization. There is a need to conduct a risk analysis and a cost-effectiveness study on intervention to improve public health and the livelihoods of the producers which are women in majority in Yamoussoukro.

## Introduction

Poor sanitation management often has a negative impact on the quality of the water that is used for urban agriculture (UA) in developing countries. The sanitation situation in Sub-Saharan Africa continues to be critical, with a high proportion of people practicing open defecation (20%). Wastewater contains a large range of contaminants from municipal, agricultural and industrial sources. Pathogens originating from this water source pose health risks to farmers and their families, communities living in proximity to wastewater irrigation and consumers of wastewater-irrigated crops (Dickin et al. 2016; Hamilton et al. 2006).

UA plays an important role in enhancing and maintaining food security worldwide. Dealing with increasing global urbanization and environmental threats, ensuring food security for poorer populations becomes a critical issue in developing countries. A recent study showed that UA appears to be a main source of household income, facilitates women’s contribution to household food availability and provides other benefits such as economic and social advancements (Poulsen et al. 2015). However, although UA practices contribute to food security, at the same time, they increase human and environmental exposure to harmful substances and microorganisms, especially in developing countries. Wastewater used in UA is usually a complex mixture of chemical and microbiological contaminants in which the presence of faecal bacteria is of significant concern (Pavione et al. 2013). In Abidjan, the economical capital of Côte d’Ivoire, an estimated 30 million m^3^ of wastewater is released each year into the environment without prior treatment (Soro et al. 2010).

The use of wastewater for irrigation is common in most UA settings and represents a potential risk for farmers (Ferrer et al. 2012). Ishii et al. have shown that using wastewater in farming activities has been a key driver of acquiring water and foodborne infections for a long time (Ishii et al. 2014). The increase in fresh vegetable consumption has led to a corresponding rise in the number of foodborne disease outbreaks linked to production (Bichai and Smeets 2013; Coulliette et al. 2013). Diarrhoea is one of the main causes of morbidity due to inadequate sanitation. An estimated 1.7 million people, among whom 90% are children of less than 5 years of age, die each day from diarrhoeal diseases (Prüss-Ustün et al. 2014). *Escherichia coli* O157:H7 and *Giardia lamblia*, two major causatives of diarrhoea, are found in the environment, food and animals and occur with inadequate sanitation or unsafe drinking water. In the Agboville area in southern Côte d’Ivoire, a prevalence of 13.9% was reported for diarrhoeal infections caused by *G. lamblia* among schoolchildren (Ouattara et al. 2010). Poor wastewater management is observed in all urban areas in Côte d’Ivoire, and is mostly related to the lack of investment in constructing sanitation infrastructures. Because of water scarcity, wastewater is used for irrigating crops in Côte d’Ivoire. Yamoussoukro, the political and administrative capital of Côte d’Ivoire, has used more than ten lakes for irrigating crops in the last decades. These lakes receive storm water as well as untreated wastewater from anthropogenic activities (Kouamé et al. 2014a; Seidl 2006). Septic tanks constitute up to 90% of the sanitation facilities in this city, and the resulting faecal sludge is discharged into the lakes. UA activities rely on water from those lakes; therefore, it is unsurprising that the local authorities have reported 3215 and 3444 cases of diarrhoea during the years 2009 and 2010, respectively. Quantitative microbial risk assessment (QMRA) is a probabilistic modelling technique, and it is the main method used to estimate the microbiological risk of infection from exposure to a microorganism (Hamilton et al. 2006).

The current study aimed to assess the microbiological risk from waterborne infections (i.e. *E. coli* O157:H7 and *G. lamblia*) linked to the exposure of urban farmers practicing UA and the consumers of raw vegetable in the city of Yamoussoukro.

## Material and methods

### Study area

**Fig. 1:**
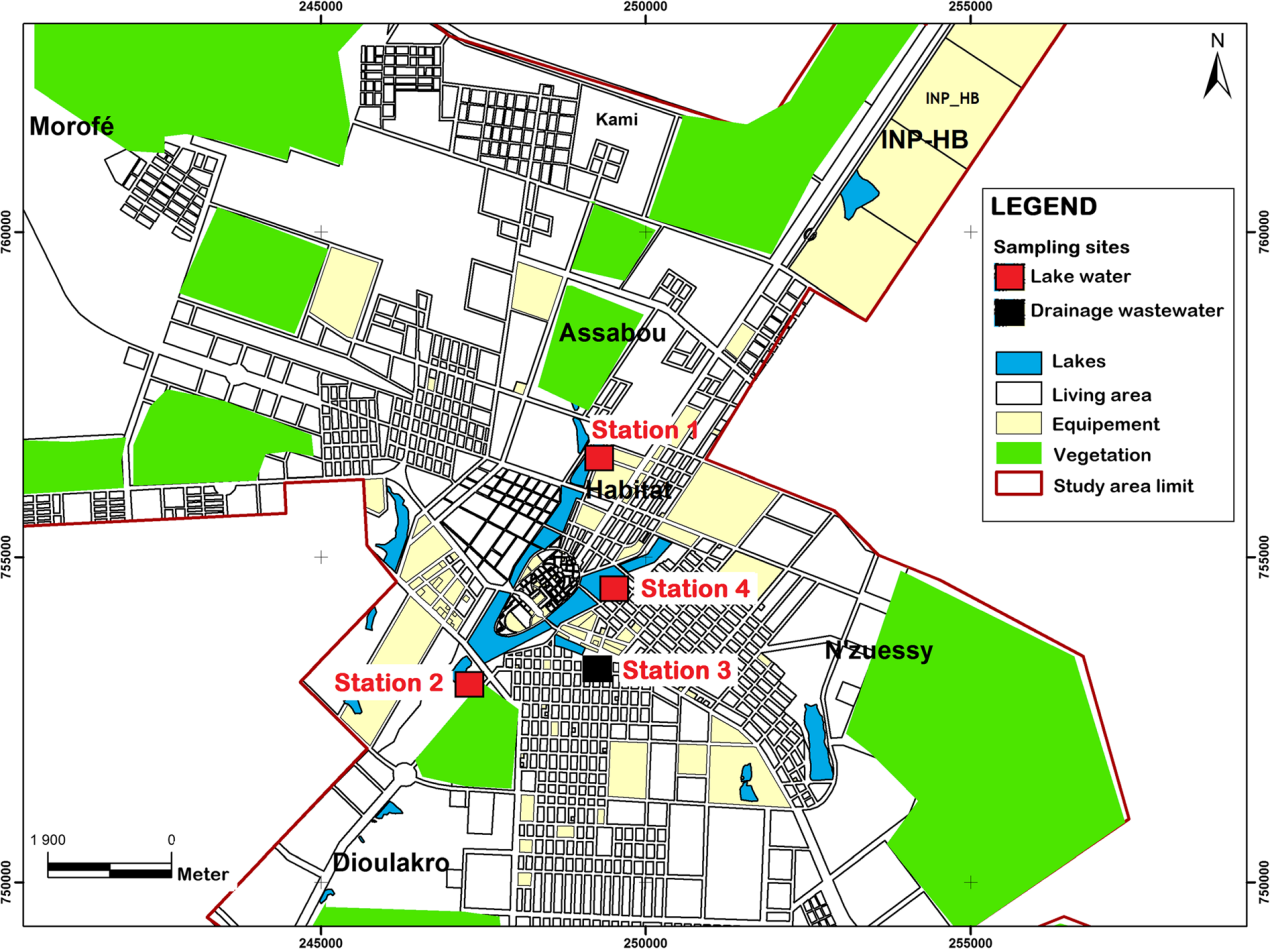
Study area and sampling sites in Yamoussoukro, Côte d’Ivoire

The city of Yamoussoukro in Côte d’Ivoire was selected as the study area at 6° 45′–6° 50′ north latitude and 5° 22′–5° 23′ west longitude (Fig. 1). The urbanized area covers 2720 ha, and a recent census conducted in 2014 estimated the urban population at 207,412 inhabitants (INS 2014). The precipitation is around 1200 mm/year, and the average annual temperature is 30 °C. Tap water is used throughout the city except in some poor neighbourhoods where water is derived from wells.

### Sample collection and laboratory analyses

The sampling and the laboratory analyses were performed according to the guidelines of the US Environmental Protection Agency (EPA) standards (EPA 2010b) during 6 months in Yamoussoukro. A total of 96 specimens of green salad (*n* = 48) and irrigation water (*n* = 48) were sampled and transported to the laboratory on ice for subsequent analyses. During the sample period, four sample points were investigated with an interval of 15 days between each sampling time point, from November 2011 to February 2015 and from May 2012 to July 2012. Lake water was sampled in sample stations 1, 2 and 4, while drainage wastewater was at sample point 3. *E. coli* O157:H7 and *G. lamblia* were selected as the indicators for faecal contamination.

After transfer to the laboratory, green salad samples were carefully crushed and 20 g were diluted in water. The analysis of pathogenic *E. coli* O157:H7 was conducted following EPA standards (EPA 2010b). Both crushed green salad and water samples were distributed into 15 tubes at 3 different concentrations (1×, 2× and 5×). After incubation at 37 °C for 24 h, the presumptive *E. coli* colonies that appeared in blue salmon colour were sub-cultured on Eosin Methylene Blue Agar for confirmation. Biocard™ EHEC kits (Ani Biotech Oy, Vantaa, Finland, Ref: 6-001-000) were subsequently used for serological confirmation of the *E. coli* O157:H7. The “Most Probable Number” of the pathogen was assessed using the US EPA method with the table of Albert Klee (EPA 2010b). *G. lamblia* cysts were studied and isolated according to the sodium acetate formalin (SAF) technique (AFNOR 2001). After 2–6 h of decantation of the 1-l crushed diluted green salad samples, the sediment was collected and diluted in 10 ml of SAF solution prior to centrifugation for 500×*g* for 10 min. The obtained precipitate was then mixed with 7 ml of NaCl solution and 3 ml of ether prior to another round of centrifugation for 500×*g* for 10 min. Debris from the side of the tube was carefully decanted, and a few drops of physiological saline to suspend the remaining sediment were added. The cysts were subsequently identified and counted with an optical microscope equipped with a ×50 magnifier.

### Microbiological risk modelling

The QMRA has been conducted as described by Haas et al. (2014). This method follows a four main step process consisting in (i) hazard identification, (ii) exposure assessment, (iii) dose–response modelling and (iv) risk characterization.

#### Hazard identification

To predict health risk in this study, we chose to analyse *G. lamblia* and *E. coli* O157:H7 which represent two well-known diarrhoea-causing organisms.

#### Exposure assessment

The aim of the exposure assessment in this QMRA was to estimate the concentrations and doses of the selected pathogens to which the affected population might be exposed to. Key parameters measured for the exposure assessment were both pathogen concentrations in green salad and irrigation water (lake water and drainage networks). The percentage of the exposed population, the frequency of exposure, and the quantity of salad consumed per day were assessed by questionnaires. The volume of water ingested and the mass of salad consumed during each type of exposure were estimated from the literature.

Two scenarios were used in the exposure assessment for this study. The first scenario concerned wastewater ingested during urban farming activities, and described the exposure dynamics when practicing UA activities using water of poor quality in Yamoussoukro. Green salad is being watered manually by farmers using contaminated wastewater. For this scenario, activity frequencies, duration and type of protection were investigated by using a questionnaire. The second scenario was related to exposure from salad vegetable consumption of which there is very little information at the population level in Côte d’Ivoire. To implement a QMRA in Yamoussoukro under these conditions, we used data from US EPA reports (EPA 2011) and published papers (EPA 2010a; Forslund et al. 2012). Equation 1 represents the ingested dose of *E. coli O157:H7* and *G. lamblia*.

1 $$ D=\left({I}_v\times {M}_c\right) $$

In this equation, *D* is the ingested dose of pathogens, *I*_*v*_ the ingested volume and *M*_*c*_ the mean concentration of the targeted pathogens. The exposed population was subsequently estimated consulting the questionnaires from those participants eating salad vegetables. It was assumed that all water sources used for irrigation in the study site have a poor quality.

A cross-sectional survey was used to assess exposure with urban farmers and at the household level including all household members above 14 years of age. Due to the non-invasive and non-intrusive nature of the study and the study questions, informed, oral consent was obtained directly from all participants prior to conducting the survey including for those aged under 18 years.

The household questionnaires were addressed preferably to the female members of the household. A total of 492 households participated in the cross-sectional survey conducted from June 2011 to July 2011. The access to sanitary facilities, clean water sources (tap or wells) and the hygiene conditions (hand and salad washing) were assessed and integrated into the risk model (Table 1). The number of people exposed and the frequency of exposure were also estimated during this process. The sample size was calculated with a precision of 5% at a 95% confidence level. Equation 2 represents the sample size calculation method.

2 $$ n=N/\left(1+{N}^{\ast }{\mathrm{e}}^2\right) $$

In this equation, *n* represents the sample size, *N* the number of inhabitants (207,412) and *e* the acceptable sampling error (5%).

**Table 1:** Input parameters with distributions and statistics for exposure assessment

Model parameter	Unit	Probability distribution function and parameter statistics			Reference
		Distribution	Mean/maximum	Minimum/S. deviation	
Volume of water ingested when farming	ml/day	Uniform	10	15	Shuval et al. (1997)
Volume of water captured by green salad vegetables	ml/g	normal	0.108	0.019	Hamilton et al. (2006)
Pathogen reduction by washing salad	log_10_ units	Pert	1	0.2	Predmore and Li (2011)
Irrigation frequency	Days per year	Normal	243.1	4.6	Surveys (2011)
Consumption frequency	Days per year	Normal	131.6	2.6	Surveys (2011)
Concentration of *E. coli O157:H7* in water	MNP/l	Log-normal	–	–	Lab. analyses (2012)
Concentration of *E. coli O157:H7* in green salad	MNP/100 g	Log-normal	–	–	Lab. analyses (2012)
Concentration of *G. lamblia* in water	Cyst/l	Log-normal	–	–	Lab. analyses (2012)
Concentration of *G. lamblia* in green salad	Cyst/20 g	Log-normal	–	–	Lab. analyses (2012)
Parameter *G. lamblia* (r)	–	Uniform	0.0198	0.02	Haas et al. (1999)
Parameter *E. coli O157:H7 (*α, β*)*	–	Uniform	*α* = 0.373	*β* = 39.71	Teunis et al. (2008)
Mass of green salad consumption	g/person/day	Normal	26.03	30.2	U.S. EPA (2009)
Exposed population	Inhabitants	Normal	–	–	Surveys (2011)

Dose–response models: two equation models of dose–response were used in the current study, including the beta-Poisson model (Mok et al. 2014) used to predict the risk linked to *E. coli* O157:H7, as described by Eq. 3.

3 $$ {P}_{\mathrm{inf}}=1-{\left(1+D/\beta \right)}^{-\alpha } $$

In this equation, *D* is the ingestion dose, whereas *α* and *β* represent the shape parameters (0.0571 and β 2.2183, respectively).

An exponential model was used for assessing the health risk linked to *G. lamblia*, as presented by Eq. 4.

4 $$ {P}_{\mathrm{inf}}=1-{\exp}^{-r\times D} $$

In this equation, *r* is 0.0199 for *G. lamblia* (Haas and Eisenberg 2001).

Risk characterization: the risk characterization combined dose–response and exposure information which results from the calculation of the annual infection probability (*P*_*y*_). The single exposure risk was assessed by performing a Monte Carlo simulation with 10,000 iterations in R software version 3.2.5 (R Development Core Team, CA, USA). The single exposure risk was assessed using a dose–response model of each pathogen model, and the exposure results were obtained from the cross-sectional surveys. The annual probability of infection associated with environmental exposure in this study was determined applying Eq. 5.

5 $$ {P}_{\inf /y}=1-{\left(1-{P}_{\mathrm{inf}}\right)}^n $$

In this equation, *P*_inf/*y*_ represents the annual probability of infection, *P*_inf_ the probability of infection for a single exposure to an ingested dose (*D*) of pathogens and *n* the number of exposure frequency, expressed in number of days per year.

The risk of infection (*P*_inf/*y*_) was subsequently converted into risk of diseases (*P*_ill_). Equation 6 presents the model of annual risk of diarrhoeal disease.

6 $$ {P}_{\mathrm{ill}}={P}_{\inf /y}\times {P}_{\mathrm{ill}/\inf } $$

In this equation, *P*_ill/inf_ is the risk of illness constant (*P*_ill/inf_ [*E. coli*] = 0.25 (Howard et al. 2006); *P*_ill/inf_ [*G. lamblia*] = 0.67 (Rose et al. 1991)).

### Statistical analyses and software

The current QMRA modelling applied a Monte Carlo simulation process. All the input parameters included in the risk model were presented as their probability distribution functions that were generated from random values (10,000 iterations) from the data collected, either from microbial analyses or household surveys. The modelling was performed using R software version 3.0.3 (Development Core Team from Vienna, Austria) with application of the R package (fitdistrplus) to fit the distribution of pathogen concentrations. The mapping was conducted using ArcGIS 10.1 to present the annual risk at sampling sites.

## Results

### Exposed population characteristics

The majority of farmers had no formal education (73.0%), and was above 21 years of age with an average age of 40 years. Among the farmers, 86.0% were males and 14.0% were females. The cross-sectional survey was conducted in 492 households representing 3307 inhabitants. Four main age classes were identified: 0–5, 6–10, 11–15 and >15 years, representing 604 (18.3%), 509 (15.4%), 452 (13.7%) and 1742 (52.7%) inhabitants. The sampled population was composed of 74.2% female and 25.8% male participants. The results showed that 82% of the population were exposed to health risks due to a lack of hygiene treatment prior to eating green salad.

### Exposure patterns in the study area

**Fig. 2:**
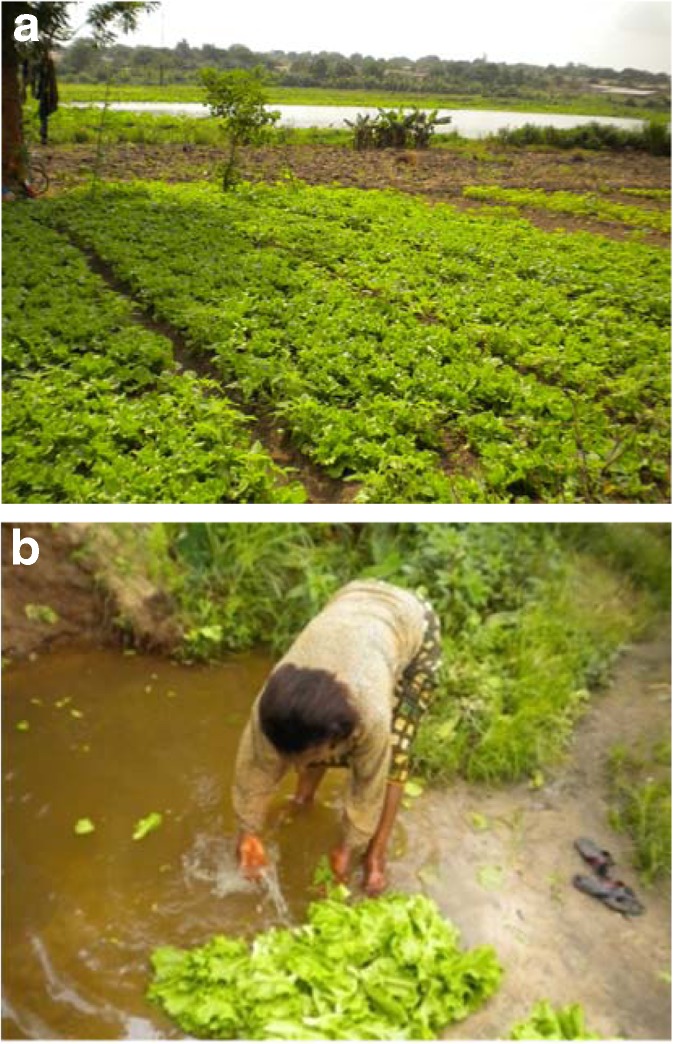
Exposure related to poor hygienic practices and irrigation water conditions in the study area. **a** Contaminated lake water used for farming activities in Yamoussoukro. **b** Exposure routes from poor hygiene of green salads

The results of the exposure cross-sectional survey conducted with 38 farmers revealed two main water sources used for irrigating green salad in the study area. The contaminated lake water and wastewater from drainage networks were used in 54 and 46%, respectively. Figure 2 represents examples of increased exposure to those contaminated water bodies that may contribute to an increased health risk of green salad consumption in Yamoussoukro. Results indicated that after the harvesting, green salad vegetables were sold to either different markets or hostels inside the city of Yamoussoukro (39%) or outside the city (61%). The results of the household cross-sectional survey suggested different types of hygiene treatment procedures of salad prior to consumption.

They include simple washing (15.7%), the use of chlorine (62.6%) and the use of alternative treatment methods, such as potassium (1.4%), vinegar (0.6%) and salt and lemon (2.4%). Only a small percentage of the sampled population (1.6%) was using salad vegetables without any prior hygiene treatment.

### Health risks linked to farming activities and salad consumption

**Fig. 3:**
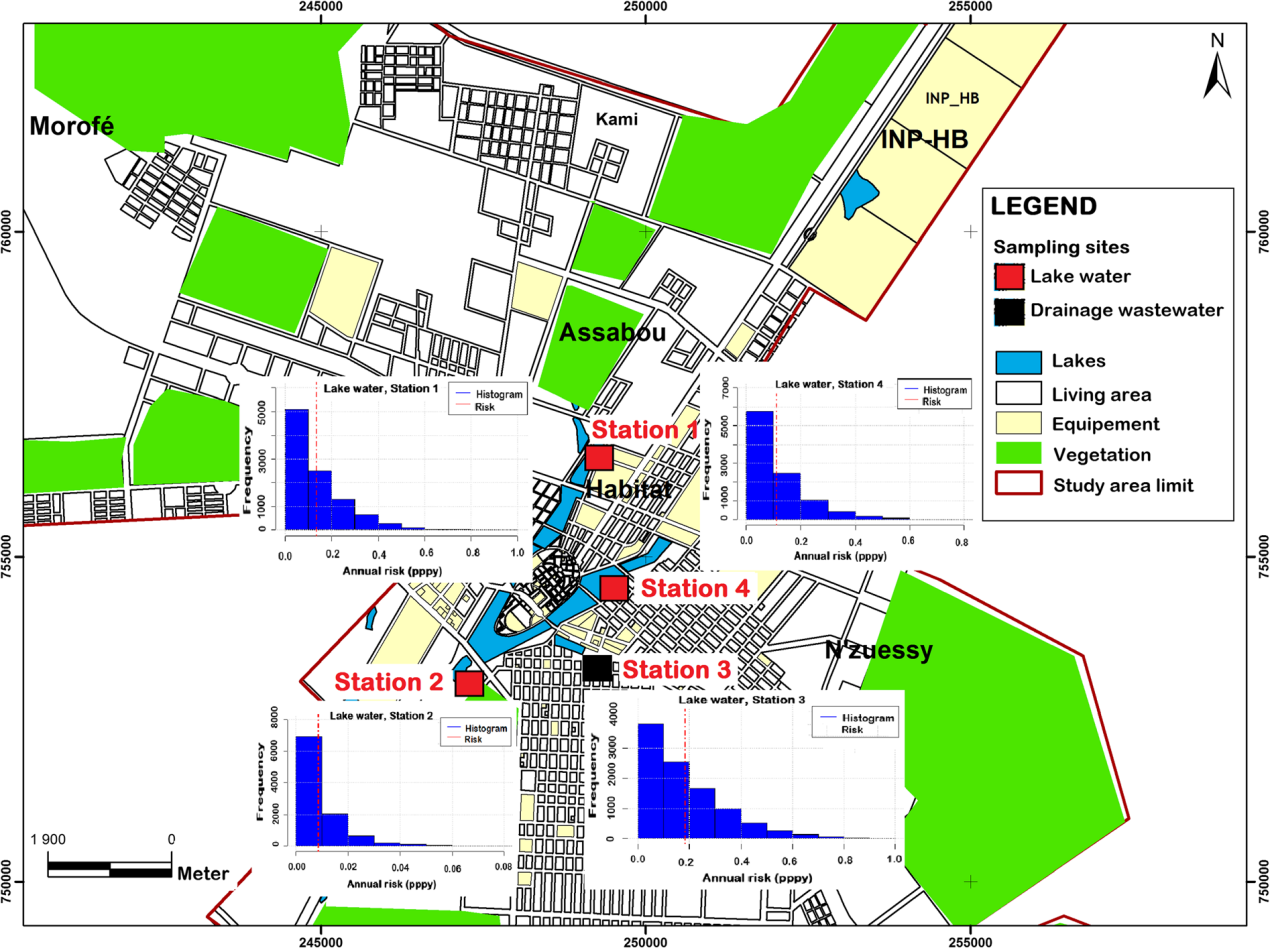
Map of annual risk linked to *E. coli* O157:H7 from irrigating with wastewater in Yamoussoukro

**Fig. 4:**
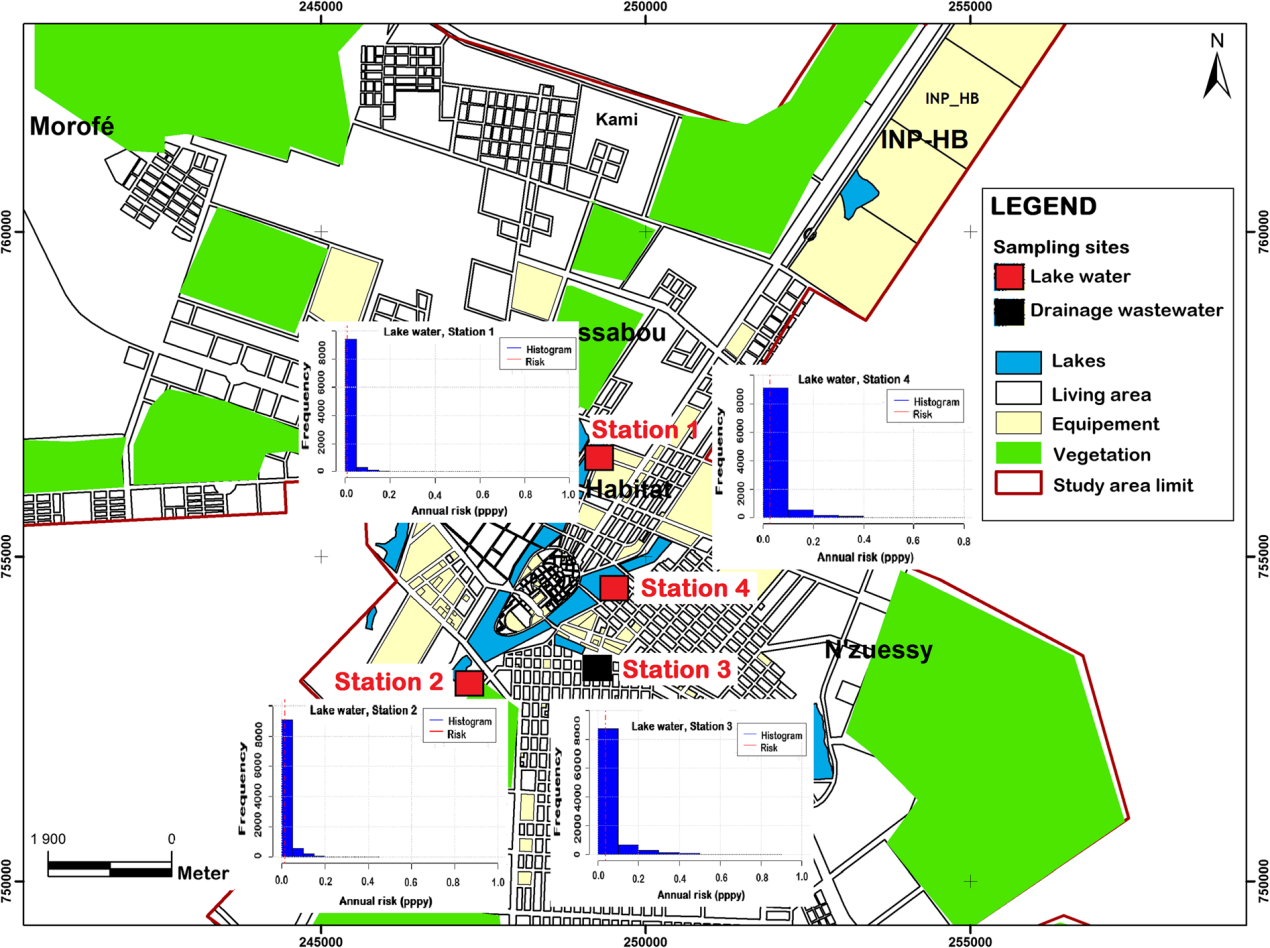
Map of annual risk linked to *G. lamblia* from irrigating with wastewater in Yamoussoukro

Concerning the probability of infection related to *G. lamblia*, the results showed that station 3 (drainage wastewater) was the biggest source of contamination during the farming activities in the city of Yamoussoukro compared with stations 1, 2 and 4 (lake water). The annual risk linked to the *G. lamblia* pathogen ranged from 0.0 to 0.36 pppy. However, a higher risk was observed in station 3, with a mean value of 0.04 pppy. The annual risk linked to *E. coli* O157:H7 was high at all four sampling sites, and ranged from 10^−9^ to 0.96 pppy. Station 3 demonstrated the highest annual risk (0.18 pppy) for this pathogen. With respect to green salad consumption, the annual risk linked to *G. lamblia* ranged from 0.0 to 1 pppy, with a mean value estimated at 0.05 pppy. A risk associated with *G. lamblia* was only demonstrated in station 3 where drainage water is used as source for irrigation. The risk linked to *E. coli* O157:H7 varied from 0.0 to 1 pppy, with a mean value of 0.93 for salad consumption at stations 1, 2, 3 and 4, whereas the maximum risk was observed at station 1 (lake water). The annual risk in this study is presented in Figs. 3 and 4.

Table 2 represents results of *P*_ill_ outcomes after using poor-quality water for irrigating green salad in the city of Yamoussoukro. With regard to the risk associated with irrigation water during farming activities, *P*_ill_ varied from 0.2 to 4.6% for *E. coli* O157:H7, and from 0.64 to 2.7% for *G. lamblia*. The highest *P*_ill_ for diarrhoeal diseases was found in station 3. Concerning salad consumption, *P*_ill_ ranged from 0.0 to 23.2% for *E. coli* O157:H7, and from 0.0 to 1.1%, for *G. lamblia*. Station 1 was more exposed to diarrhoeal diseases according to the *P*_ill_ of *E. coli* O157:H7.

**Table 2:** Probability to ill (*P*_ill_) from *E. coli* O157:H7 and *G. lamblia* by irrigation and salad consumption

Scenarios	Pathogens	1	2	3	4
1 (Farming activities)	*E. coli* O157:H7	3.40%	2.85%	4.57%	0.22%
	*G. lamblia*	0.64%	1.83%	2.66%	0.95%
2 (Salad consumption)	*E. coli* O157:H7	23.25%	0.00%	21.25%	12.25%
	*G. lamblia*	0.00%	0.00%	1.01%	0.00%

## Discussion

The current study showed that contaminated lake water and wastewater from drainage were used for farming activities in Yamoussoukro at 54 and 46%, respectively. Moreover, it was demonstrated that those water sources were contaminated with *E. coli* O157:H7 across all sample sites, and that half of the samples sites were, in addition, contaminated with *G. lamblia*. The United Nations Food and Agriculture Organization has reported that the agricultural sector is the largest user of water and wastewater globally, accounting for approximately 70% of water use worldwide (FAO 2011), which illustrates the potential scale of this health risk.

The current result in this Côte d’Ivoire-based study showed that the proportion of exposed farmers (46%) due to the use of wastewater is higher than those observed in equivalent studies in Accra (37%), but lower than Kenya (53%) and Zimbabwe (70%) (Antwi-Agyei et al. 2016). The relevant exposure routes identified during farming activities in Yamoussoukro were use of poor-quality water, the farmer’s hygiene precautions and the microbiological quality of post-harvest products. A recent study conducted in Yamoussoukro has demonstrated, indeed, how poor water quality management and wastewater use in urban agriculture can negatively impact on population health (Kouamé et al. 2014b). In the same city, diarrhoea was associated with poor sanitation, whereas households with dry latrines were found to bear a higher risk of infection compared with households that rely on latrines with septic tanks (Kouamé et al. 2014b). However, studies assessing the health risks linked to wastewater and poor sanitation in urban areas in developing countries are not limited to Yamoussoukro or Côte d’Ivoire. In Kenya, Mali and India, it has been shown that the prevalence of diarrhoeal disease transmission for children is related to the number of households that are sharing a latrine (Baker et al. 2016). In Ethiopia, the negative impact of poor sanitation, unsafe water supply and inadequate personal hygiene was demonstrated in the dynamics of diarrhoea occurrence (Gebru et al. 2014). The microbiological contamination in the current study conducted in Yamoussoukro seems to follow a similar pattern when compared with other urban areas in developing countries.

The improvement of water quality for farming activities is an important aspect in the overall strategy of reducing waterborne and foodborne diseases owing to the close link between the major microbiological contaminations and the use of raw wastewater. In-depth assessment of the association between poor sanitation, hygiene conditions and food security by analyzing the resilience of water, sanitation and hygiene systems to hazards can contribute to the reduction of health risks (Johannessen et al. 2014). The improvement of hygiene during farming activities and green salad harvest could contribute to reduce waterborne diseases in Yamoussoukro.

At the household level, the key exposure routes identified in Yamoussoukro were the lack of hand and green salad washing prior to salad consumption and the presence of poor hygiene conditions. Our study showed that only 15.7% of all household members assessed practiced simple washing of green salad prior to consumption, and that 62.6% of this population used chlorine as second treatment. The decontamination of green salad by applying hygiene measures is a strong determinant in the reduction of health risks. The microbiological quality of an agricultural food product is influenced by many factors including the type of vegetable, the temperature, humidity and exposure to sunlight during cultivation, the application of irrigation water and post-harvest handling (Antwi-Agyei et al. 2015).

The results of the QMRA confirmed the poor wastewater quality used for irrigation in Yamoussoukro. AU practices increased the microbiological exposure risk of the two targeted groups of individuals—urban farmers and the inhabitants of an average household. Concerning the quality of water for irrigating vegetables, the annual risk is estimated at 0.36 for the pathogen *G. lamblia* and 0.20 pppy for *E. coli* O157:H7. The probability to ill (*P*_ill_) from salad consumption is estimated at 1.0% for *G. lamblia* and 23.2% for *E. coli* O157:H7. The results showed that the annual risks of infection from farming activities and salad vegetable consumption were higher than the tolerable limit of risk defined by the World Health Organization as 10^−4^ for irrigating water quality (WHO 2006) and 10^−6^ for consumption (Asano 2007; WHO 2006). Similarly high levels of risk linked to *E. coli* O157:H7 and *G. lamblia* were found in Abidjan, where the reuse of wastewater and lagoon water was investigated (Yapo et al. 2014). However, the annual risk from *G. lamblia*, identified in Yamoussoukro, was lower compared with findings in Thailand, where the risk of *G. lamblia* infection from eating vegetables linked to irrigated wastewaters was 100% (Ferrer et al. 2012).

The *P*_ill_ for diarrhoeal diseases related to the use of wastewater in farming activities was found to be very high in Yamoussoukro (estimated at 4.6% for *E. coli* O157:H7, and 0.64% for *G. lamblia*). As in many cities in West Africa, the use of low-quality wastewater in urban agriculture increases exposure routes to infective agents and, hence, increases the population health risk. The results obtained in Yamoussoukro showed that irrigation water for green salad is contaminated with faecal bacteria as indicated by the presence of *E. coli* O157:H7 and *G. lamblia*. These pathogens represented a threat for farmers as well as consumers. Similar observations have been made in a study conducted in Accra (Ghana) by identifying risk factors for product contamination at different entry points of the food chain. After analyzing 500 samples of product and ready-to-eat samples of green salads, the author showed that over 80% of the product samples were contaminated with *E. coli*, and that the risk factors identified were irrigation water, storage and hygiene (Antwi-Agyei et al. 2015). Moreover, analysis of different contexts of QMRA implementation in urban settings of developing countries shows that the health risks linked to farming practices could occur by use of both raw wastewater and partially treated wastewater.

The implementation of QMRA in the current setting of Yamoussoukro has shown that the use of raw wastewater for irrigating vegetables represents a potential microbiological health hazard. We demonstrate the importance of the development of mitigation strategies in order to handle health conditions, especially concerning farming activities and hygiene improvements, and offer a base for decision-making.

## Conclusions

This study highlights the exposure routes and the risks of infection with waterborne diseases linked to the poor water quality used for farming activities in the context of water scarcity and food security. Facing this dilemma, farmers have to be sensitized about the potential occupational health threat when using lake waters and wastewaters from drainage networks during UA practices in Yamoussoukro. Equally, the population needs to be educated about hygiene practices before consuming salad vegetables. Innovative water treatment infrastructures have to be implemented in order to treat wastewater pollutants before agricultural use and to optimize the current public health situation.
